# Higher Apparent Gas Transfer Velocities for CO_2_ Compared to CH_4_ in Small Lakes

**DOI:** 10.1021/acs.est.2c09230

**Published:** 2023-05-30

**Authors:** Gustav Pajala, David Rudberg, Magnus Gålfalk, John Michael Melack, Sally Macintyre, Jan Karlsson, Henrique Oliveira Sawakuchi, Jonathan Schenk, Anna Sieczko, Ingrid Sundgren, Nguyen Thanh Duc, David Bastviken

**Affiliations:** † Department of Thematic Studies − Environmental Change, Linköping University, Mäster Mattias väg, Linköping 58183, Sweden; ‡ Department of Ecology, Evolution and Marine Biology, University of California, UCEN Rd, Santa Barbara, California 93117, United States; § Earth Research Institute, University of California, Lagoon Rd, Santa Barbara, California 93106, United States; ∥ Marine Science Institute, University of California, Isla Vista, Santa Barbara, California 93117, United States; ⊥ Climate Impacts Research Centre, Department of Ecology and Environmental Sciences, Umeå University, Linnaeus väg 6, Umeå 90736, Sweden

**Keywords:** carbon dioxide, methane, lake, gas transfer, greenhouse gas, piston velocity

## Abstract

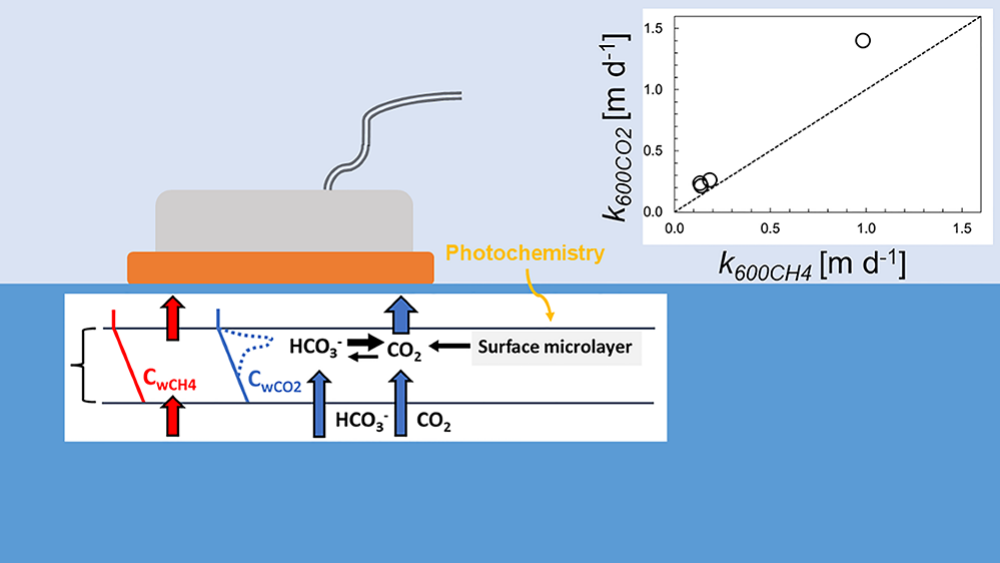

Large greenhouse gas emissions occur via the release of carbon dioxide (CO_2_) and methane (CH_4_) from the surface layer of lakes. Such emissions are modeled from the air–water gas concentration gradient and the gas transfer velocity (*k*). The links between *k* and the physical properties of the gas and water have led to the development of methods to convert *k* between gases through Schmidt number normalization. However, recent observations have found that such normalization of apparent *k* estimates from field measurements can yield different results for CH_4_ and CO_2_. We estimated *k* for CO_2_ and CH_4_ from measurements of concentration gradients and fluxes in four contrasting lakes and found consistently higher (on an average 1.7 times) normalized apparent *k* values for CO_2_ than CH_4_. From these results, we infer that several gas-specific factors, including chemical and biological processes within the water surface microlayer, can influence apparent *k* estimates. We highlight the importance of accurately measuring relevant air–water gas concentration gradients and considering gas-specific processes when estimating *k*.

## Abstract 2

Higher gas transfer velocities for CO_2_ than CH_4_ in lakes challenge previous results and commonly made assumptions and highlight the importance of gas-specific transport in aquatic greenhouse gas exchange.

## 1 Introduction

Lakes cover less than 2% of the terrestrial surface area^1^ and are estimated to emit carbon dioxide (CO_2_) and methane (CH_4_) in notable amounts with respect to the continental greenhouse gas exchange.^2,3^ Transport of dissolved gases across the water surface to the atmosphere constitutes the main pathway for lake emission of CO_2_ and is also a major flux pathway for CH_4_. The exchange of dissolved gases (Flux (*F*); mol m^–2^ d^–1^; units provided here and below are examples) between air and water depends on the concentration gradient expressed as the difference between the surface water gas concentration (*C*_w_; mol m^–3^) and the gas concentration in equilibrium with the air (*C*_air_; mol m^–3^), and the gas transfer velocity (*k*; m d^–1^), according to eq 1,
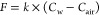
1

Flux measurements by floating chambers (FCs) or eddy covariance have been used together with measurements of *C*_w_ and *C*_air_ to calculate *k* from eq 1, yielding local apparent *k* estimates.^4−7^ There has also been several attempts to develop general *k* models, predicting *k* from external drivers.^8−11^ A common assumption is that *k* can be converted between gases of interest if the key thermodynamic properties of the dissolved gases (i.e., the ratio of the kinematic viscosity and mass diffusivity, expressed through the “Schmidt number”) are considered.^10^ Usually, *k*-values are normalized to a Schmidt number of 600 (*k*_600_), corresponding to *k* for CO_2_ at 20 °C. This Schmidt normalization procedure was derived for ideal well-defined *k* values considered predictable from fundamental physical principles. However, the apparent *k* used for estimating gas fluxes in nature is influenced by many factors,^12^ such as turbulence within the water column,^9,11^ physical mechanisms,^13^ biological and chemical mechanisms,^14,15^ or methodological uncertainties,^16,17^ that all determine how well-defined and accurate the apparent *k* becomes.

Recently, mismatches between apparent *k*_600_ for CO_2_ and CH_4_ in lakes and other aquatic systems have been observed.^4,5,7,13,18−20^ In some cases, higher *k*_600_ were found for CH_4_ than for CO_2_, attributed to CH_4_ microbubble formation and transport.^13,18^ This explanation is linked to the assumption that gases with low solubility form or enter microbubbles that move faster across the water surface boundary layer than dissolved gases, but its importance for CH_4_ depends on circumstantial indications. In one other case, higher *k*_600_ for CO_2_ than for CH_4_ were observed, which could be attributed to hydrodynamic and processes in the surface boundary layer that favors production and transport of CO_2,_^7^ but the importance and general validity of this explanation for the *k*_600_ difference between CO_2_ and CH_4_ is also unclear. Further, for both these types of observations, there may be other, not yet widely discussed, potential explanations (details in the Discussion).

The incompatibility between apparent *k*_600_ for CO_2_ and CH_4_ generates questions regarding the assumption that apparent *k* determined for one gas can be easily converted to *k* for another gas via physical properties. If apparent *k* estimates are not directly comparable across gases, the general use of models to determine *k* beyond the physicochemical domains and the specific gas(es) represented by the measurements behind the model can be questioned. All of this could undermine the global estimates of gas emissions which are dependent on such models, and the interpretation of apparent *k* and comparability of apparent *k*_600_ among gases are challenged by the above discrepancies. In this study, we investigate differences in apparent *k*_600_ for CO_2_ and CH_4_ in four lakes, with different nutrient and dissolved organic carbon concentrations and discuss possible mechanisms for gas-specific variations in apparent *k*_600_. We hypothesized contrasting *k*_600_ for CO_2_ and CH_4_ and that the *k*_600_ ratio between CO_2_ and CH_4_ would differ depending on lake characteristics (trophic status).

## 2 Materials and Methods

### 2.1 Study Areas

Empirical data were collected in four lakes (Table 1): Övre Björntjärn (OBJ; Aug. 21–24, 2012), Sörsjön (SOR; Oct. 31, 2019), Bolen (BOL; Nov. 1, 2019), and Södra Teden (SOD; Nov. 1, 2019). OBJ is a small boreal and humic lake located in northern Sweden with an inlet draining a catchment of mires and coniferous forests.^11,21^ SOR, BOL, and SOD are oligo-, meso-, and eutrophic lakes, respectively, located in southern Sweden. Their catchments consist of coniferous and deciduous forests, and BOL and SOD are adjacent to small urban areas (about 500 inhabitants).

**Table 1:** Lake Characteristics for OBJ, SOR, BOL, and SOD^a^

system	OBJ		SOR		BOL		SOD	
coordinates	N	E	N	E	N	E	N	E
	64.122	18.785	58.723	16.084	58.781	16.154	58.339	16.023
area (km^2^)	0.05		0.21		0.48		0.69	
average depth (m)	4		4.4		7.2		1.8	
maximum depth (m)	9		9		14		3.4	
pH	4.0		6.8		7.2		7.8	
DOC (mg L^–1^)	22		21.5		10.9		14.7	
TN (mg L^–1^)	0.5		0.95		0.6		1.5	
TP (μg L^–1^)	19		10		12		323	
trophic/humic status	mesotrophic/humic		oligotrophic/humic		mesotrophic		eutrophic	

a Water chemistry data for OBJ from Klaus et al.^21^ and MacIntyre et al.^11^ Water chemistry for SOR, BOL, and SOD was sampled near the surface (0.1–0.5 m depth) during the ice-free season in 2020. DOC, TN, and TP denote dissolved organic carbon, total nitrogen, and total phosphorous, respectively. Trophic status is based on calculations from Carlson,^22^ using TP.

### 2.2 Measurements

#### 2.2.1 Gas Flux

Gas fluxes were measured using FCs, similar to those used in Natchimuthu et al.^23^ The FCs consisted of plastic buckets with an opening of 0.062–0.075 m^2^ facing the water surface and volumes of 5.4–8.6 L, depending on the model used. FCs were covered with reflective aluminum tape to minimize internal heating and had floats attached to the outer edges such that the edges penetrated 2–3 cm below the water surface. In previous studies, this FC design yielded flux estimates similar to other methods that do not interfere with the water surface.^24,25^

In OBJ, 5–6 FCs were deployed simultaneously for 15–30 min. FC deployments were made 1–3 times per day over 4 days, usually between 11:00 and 14:00, and one day between 20:45 and 21:00, yielding 37 individual FC deployments. Sampling of 30 mL gas from inside the chamber was made every 10–30 min during 30-minute-long deployments by syringe sampling via a 50 cm long polyurethane tube (inner and outer diameter of 3 and 5 mm, respectively) at the top of each chamber. This resulted in 2–4 samples for each FC deployment (13 deployments with two samples, 11 deployments with three samples, and 8 deployments with four samples). Samples were analyzed within 24 h on a greenhouse gas analyzer (LGR DLT 100, Los Gatos Research Inc. USA) equipped with a custom-made syringe injection system. Regressions between FC headspace concentrations and time generated the rate of gas accumulation in each FC (ppm d^–1^). Data were discarded when the regression *R*^2^ for gas accumulation of either CO_2_ or CH_4_ was below 0.9 or if the data indicated leakage or sample handling errors (5 out of 37 measurements were discarded in total). After processing the data, 32 pairs of CO_2_ and CH_4_ accumulation rates were obtained.

In SOR, BOL, and SOD, one FC was deployed for repeated continuous measurements of gas flux for 10–16 min. Measurements were made by connecting the top of the FC to two polyurethane tubes, which were inserted to the inlet and outlet of an ultra-portable greenhouse gas analyzer (UGGA; Los Gatos Research Inc. USA). The FC connected to the UGGA was left floating freely on the water surface, see Figure S1. Between each measurement, the FC was lifted for ventilating it, allowing concentrations of CO_2_ and CH_4_ to reach background atmospheric concentrations. This provided data for a total of 16 individual FC deployments: seven in SOR, four in BOL, and five in SOD (no measurements were discarded). At least the first 60 s of gas accumulation was removed to allow time for mixing between the FC headspace and the UGGA measurement cell. Ten-second (0.1 Hz) data were used in regressions for gas accumulation rates (ppm d^–1^) for a time interval set as the minimum time needed for a gas concentration increase rate with an *R*^2^ > 0.7 to be established (between 40 and 100 s of data; *R*^2^ threshold slightly lower than for OBJ above to account for the random noise in gas spectrometry). Gas fluxes were calculated from the gas accumulation rates in the chambers and the ideal gas law according to Rudberg et al.^26^ (See Supporting Information, Text S1 for detailed information.)

#### 2.2.2 Dissolved CO_2_ Concentration

In SOR, BOL, and SOD, *C*_wCO2_ was sampled through a headspace extraction technique (e.g., Cole et al.^27^). Near-surface water was collected with a Ruttner sampler deployed horizontally at ∼0.1 m. The outlet tube of the Ruttner sampler was inserted at the bottom of a 1.2 L plastic bottle and water was transferred, overflowing the bottle with at least two times the bottle volume. Then, the bottle was capped with a rubber stopper pierced by a long and a short polyurethane tube (inner and outer diameters of 3 and 5 mm), reaching the bottom of the bottle and the end of the stopper, respectively, each connected to closed 3-way luer-lock valves at the outer end. After connecting a 60 mL syringe (Becton–Dickinson) filled with atmospheric air to the short tube and connecting an empty 60 mL syringe to the long tube, air was pushed into the bottle via the short tube, while the added pressure pushed out a similar amount of water through the long tube, filling the empty syringe. The bottle was shaken for two minutes to equilibrate gases between the headspace and the water. The equilibrated headspace air was then extracted by the inverse procedure and transferred to evacuated vials. Separate air samples were collected to correct for initial CO_2_ in the headspace.

For 21 of the flux measurements in OBJ, surface water gas concentrations of CO_2_ (*C*_wCO2_) were derived from separate chambers (here termed as equilibration chambers) equipped with CO_2_ sensors (Senseair K33 ELG, Sweden) according to Bastviken et al.^28^ Equilibration chambers were deployed for 24 h prior to flux measurements, allowing CO_2_ concentrations in the chamber headspace to equilibrate with the water underneath. Equilibration chambers were placed next to the FCs used for flux measurements to minimize differences in spatial variability between measurements of CO_2_ flux and *C*_wCO2_. To make sure that the equilibration chambers provided reliable measures for *C*_wCO2_, snapshot samples for *C*_wCO2_ were collected using a headspace extraction technique described above. Parallel measurements with the equilibrated chambers and the headspace extraction technique of water samples differed by less than 5%.^11^ For the additional 11 flux measurements in OBJ, CO_2_ concentrations were measured with a common syringe headspace extraction technique by extracting 40 mL of water at ∼0.1 m depth and 20 mL of air with a 60 mL syringe. The sample was equilibrated by shaking, and the resulting headspace gas was transferred to a dry syringe and analyzed within 120 min. Separate air samples were collected to correct for initial CO_2_ in the headspace.

#### 2.2.3 Dissolved CH_4_ Concentration

Dissolved CH_4_ (*C*_wCH4_) was sampled using two approaches: for SOR, BOL, SOD, and for 21 of the flux measurements in OBJ, water was sampled next to the FCs at ∼0.1 m depth using a 10 mL plastic syringe (Becton–Dickinson). The sampled volume was adjusted to 5 mL and transferred to a 22 mL N_2_-filled glass vial (Agilent) containing 0.1 mL 85% H_3_PO_4_ and sealed with a butyl rubber stopper and aluminum crimp. The initial overpressure of N_2_ was removed prior to injection to adjust the vial pressure to ambient conditions. The acid preserved the sample and allowed the storage of CH_4_ until analysis.

For the remaining 11 flux measurements in OBJ, water concentrations were sampled similarly to what was described in Section 2.2.2 for the additional 11 dissolved CO_2_ concentration samples, with a common syringe headspace extraction technique.

#### 2.2.4 Atmospheric Pressure and Wind Speed

At OBJ, atmospheric pressure and wind speed were measured at 10 m height from a meteorological station located 300 m north-east of the lake using an Onset S-WCA-M003 and an Onset S-BPB-CM50, respectively.^18^ For SOR, BOL, and SOD, atmospheric pressure and wind speed at 10 m were obtained from the Swedish Meteorological and Hydrological Institute (SMHI) MESAN model. The model interpolates measurements from nearby weather stations combined with a meteorological model to estimate hourly means on a 2.5 × 2.5 km grid.^29^ Although MESAN estimates wind speed at 10 m height, previous results from two lakes showed reasonable agreement between hourly MESAN values and wind speed measured at lake level (*R*^2^ = 0.65–0.74).^26^

### 2.3 Analysis of Dissolved Gas

The samples in vials from all lakes were analyzed by gas chromatography (Agilent 7890) with a Poropak Q column and FID detection connected to a headspace autosampler (Agilent 7697 headspace sampler). Samples in syringes from OBJ were analyzed in the field on the Los Gatos DLT100 greenhouse gas analyzer as described above. Water concentrations were calculated using the partial pressures (the measured mixing ratio times the barometric pressure) combined with (i) the ideal gas law to calculate the amount of the gas in the headspace (*n*_h_; mol) and (ii) the temperature adjusted Henry’s law^30^ to calculate the residual gas in the water within the vial/syringe (*n*_aq_; mol). The sum of *n*_h_ and *n*_aq_, after subtracting the background target gas amount in the gas forming the headspace (N_2_ in vials and atmospheric air in the syringe extractions), divided with the volume of the water sample extracted yielded target gas concentrations. For CO_2_, we accounted for shifts in the carbonic acid equilibrium during equilibration according to Koschorreck et al.^17^ and Rudberg et al.^26^

### 2.4 Calculating *k*_600_

We used paired gas flux and concentration measurements of CO_2_ and CH_4_ to derive *k* from eq 1. These values were then converted to a standardized *k* with the same Schmidt number of 600 (*k*_600_), i.e., the relationship between the kinematic viscosity of water divided by the diffusion coefficient of gas normalized to CO_2_ at 20 °C,^10^ to allow comparison of gases^12^ according to eq 2:
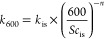
2where *k*_is_ and Sc_is_ are *k* and Schmidt numbers in situ, 600 is the reference Schmidt number, and *n* is a variable that is linked to the roughness of the water surface. Similar to other studies^4−7^ and according to Jähne et al.^12^ and Liss and Merlivat,^31^ we have used a value for *n* of ^2^/_3_ and ^1^/_2_ in conditions when wind speeds, either measured at 10 m or corrected to a height of 10 m, were below and above 3.6 m s^–1^, respectively.

### 2.5 Statistical Analysis

Parameters and *R*^2^, adjusted to the number of observations (*n*) and predictor variables (*p*), according to eq 3, were derived from Ordinary Least Squares regression for linear relationships and from direct curve-fitting in Python v.3.7 (scipy.optimize.curve_fit) for exponential relationships, where the latter estimates the parameters without log-transformation to not bias large *x*-values.
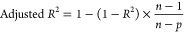
3

Adjusted *R*^2^ values according to eq 3 are in the following sections, including text and figures, referred to as *R*^2^. Since *k*_600_ data violated terms for parametric tests by not being normally distributed, the non-parametric Wilcoxon signed-rank (S-R) test and the Kruskal–Wallis analysis of variance rank test were performed in IBM SPSS statistics 28 to test for differences in *k*_600_ values for CO_2_ and CH_4_. We considered *p*-values below 0.05 as statistically significant to reject null-hypotheses in statistical tests and regressions.

## 3 Results

All surface waters were supersaturated with CO_2_ and CH_4_, with water to air concentration ratios (*C*_w_/*C*_air_) ranging from 3.0 to 5.5 for CO_2_ and from 95 to 335 for CH_4_. *C*_wCO2_ and *C*_wCH4_ ranged from 70 to 118 and from 0.39 to 1.38 μmol L^–1^, respectively. This variability was mostly due to differences between lakes whereas within-lake variability in *C*_wCH4_ and *C*_wCO2_ was small (within-lake CV: 2–31% and 2–11% for *C*_wCH4_ and *C*_wCO2_, respectively; Figure S2a). The measured fluxes ranged from 4 to 132 mmol m^–2^ d^–1^ and 0.02 to 0.98 mmol m^–2^ d^–1^ for CO_2_ and CH_4_, respectively, with the highest fluxes observed in OBJ (Figure S2b). Fluxes of CO_2_ and CH_4_ were highly related (*p* < 0.001, *R*^2^ = 0.91; Figure S2b). The relationship was mainly driven by OBJ data where a greater flux range was observed, but it was also significant when OBJ data were excluded (*p* < 0.05, *R*^2^ = 0.43).

**Figure 1:**
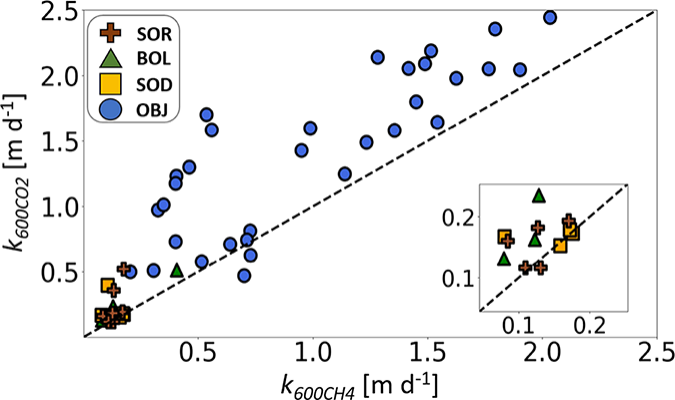
Plot of apparent gas transfer velocities normalized to a Schmidt number of 600, derived for CO_2_ (*k*_600CO2_) and CH_4_ (*k*_600CH4_) for lakes OBJ (blue circles), BOL (green triangles), SOD (orange squares), and SOR (brown crosses). The dashed line shows a 1:1 relation, where values above and below this line denote higher *k*_600_ for CO_2_ and CH_4_, respectively. The inset panel shows the smallest *k*-values and the dashed 1:1 line for clarity.

Values of *k*_600_ ranged between 0.12 and 2.24 m d^–1^ for CO_2_ and between 0.08 and 1.84 m d^–1^ for CH_4_ (Figure 1), with an overall mean and median *k*_600_ ratio (*k*_600CO2_/*k*_600CH4_) of 1.68 ± 0.77 (mean ± 1 standard deviation) and 1.37, respectively, with only small differences in mean values between the lakes (OBJ = 1.67 ± 0.75 m d^–1^, SOR = 1.72 ± 0.76 m d^–^1, BOL = 1.52 ± 0.24 m d^–1^, and SOD = 1.76 ± 1.07 m d^–1^). This confirms the first part of our hypothesis that *k*_600_ for CO_2_ and CH_4_ would differ (*p* < 0.001). However, the second part of our hypothesis, that lake trophic status would influence the CO_2_/CH_4_*k*_600_ ratio, was not verified as this ratio was similar among the studied lakes (*p* > 0.05).

**Figure 2:**
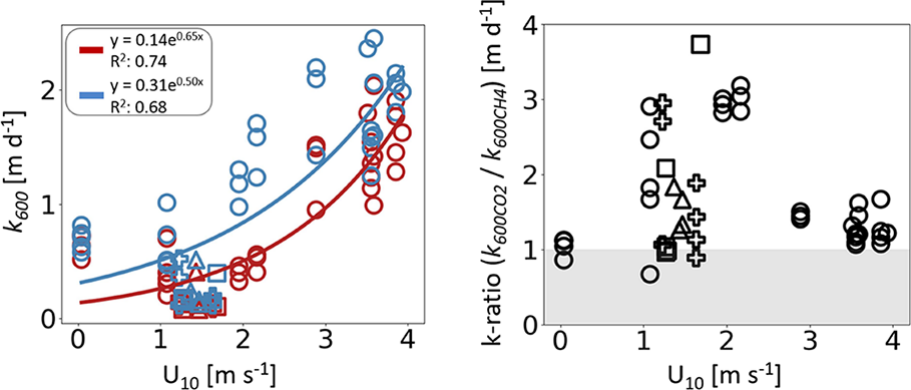
Regressions between wind speed at 10 m height (U_10_) and (a) apparent gas transfer velocities normalized to Schmidt number 600 for CH_4_ (*k*_600CH4_; red) and CO_2_ (*k*_600CO2_; blue), and (b) apparent *k*_600_-ratio (*k*_600CO2_/*k*_600CH4_). Different symbols represent different lakes: OBJ (circles), BOL (triangles), SOD (squares), and SOR (crosses). Gray area highlights where *k*_600CO2_ < *k*_600CH4_.

We observed that *k*_600CO2_ and *k*_600CH4_ were exponentially related to wind speed, which ranged from <0.4 to 3.9 m s^–1^ in OBJ and from 1.2 to 1.7 m s^–1^ elsewhere (*R*^2^ = 0.69 to 0.75; Figure 2a; the relationship is primarily generated by OBJ data, where the wind speed range was greatest). There was no clear unidirectional relationship between wind speed and the *k*_600_ ratio for CO_2_ and CH_4_ (Figure 2b). The *k*_600_ ratio was negatively and positively linked to CO_2_ and CH_4_ saturation (gas concentration in the water divided by the theoretical concentration in equilibrium with the atmospheric partial pressure), respectively (Figure S3). Such trends were weak, yet significant considering all lakes combined (CO_2_; *p* < 0.05, *R*^2^ = 0.12, CH_4_; *p* < 0.01, *R*^2^ = 0.20), and driven mainly by the OBJ lake data (CO_2_; *p* < 0.001, *R*^2^ = 0.32, CH_4_; *p* < 0.001, *R*^2^ = 0.64).

## 4 Discussion

### 4.1 Comparisons with Previous Studies

Our mean and median apparent *k*_600_ ratios for lakes, showing higher *k*_600CO2_ by a factor of 1.7 and 1.4 compared to *k*_600CH4_, are consistent with one other study, suggesting that apparent *k*_600CO2_ exceeded *k*_600CH4_.^7^ More specifically, Rosentreter et al.^7^ observed mean and median values for *k*_600_ ∼ 2.8 and 1.6 times greater for CO_2_ compared to CH_4_ in a mangrove estuary. In contrast, observations from some other studies report higher apparent *k*_600_ for CH_4_ than for CO_2_ (Table 2). Prairie and del Giorgio,^13^ McGinnis et al.,^18^ and Rantakari et al.^19^ found higher *k*_600_ for CH_4_ in 90–100% of their measurements, compared to 10 and 17% of the measurements in our study and Rosentreter et al.,^7^ respectively. These mixed results indicate variability in *k*_600_ between gases, among systems and conditions for reasons not yet understood.

**Table 2:** Examples of Results from Studies Comparing *k*_600_ for CO_2_ and CH_4_ in Order of Lower to Higher *k*_600CO2_/*k*_600CH4_ Ratios^a^

source	systems	*C*_aq_ method		*k*_600_ (m d^–1^)				
		CO_2_	CH_4_	CO_2_		CH_4_		CO_2_/CH_4_
				min	max	min	max	
McGinnis et al.^18^	temperate lake	Eq	Eq	1.97	7.2	3.7	22	0.4
Paranaíba et al.^6^	three tropical reservoirs	Eq	Eq	0.1	7.9	0.2	19.1	0.4
Prairie and del Giorgio^13^	boreal reservoir and lakes	Eq	Hs	0.1	9.3	0.1	25	0.43
Rantakari et al.^19^	two boreal lakes	Hs	Hs	0.5	3.4	1.1	14.5	0.56
Rosentreter et al.^20^	six mangrove estuaries	Eq	Eq	1	24	1	28	0.83
Beaulieu et al.^5^	temperate river	Hs	Hs	0.2	13.7	0.4	16.8	< 1
Guérin et al.^4^	tropical reservoir	Hs	Hs	0.05	1.9	0.1	2.2	1.16
This study	four boreal lakes	Hs, Eq^b^	Hs	0.1	2.4	0.1	2	1.68
Rosentreter et al.^7^	mangrove river estuary	Eq	Hs	0.3	48	0.02	16	2.78

a Eq and Hs denote measurements with flow through equilibrator and headspace extraction techniques, respectively, for surface water concentrations. The equilibrated chambers technique includes headspace extraction in floating chambers allowed to equilibrate.

b Equilibrated chambers were used in 21 of the samples from OBJ, and headspace extraction was used in the remaining 11 samples from OBJ and in SOR, BOL, and SOD.

The results seem to indicate a hump-shaped relationship between the *k*_600_ ratio and wind speed (Figure 2b). The results indicate that differences in *k*_600_ between CO_2_ and CH_4_ can be substantial even at low wind speeds in both estuaries^7^ and lakes and could be larger at intermediate wind speeds for unknown reasons. If correct, it can be speculated that some of the mechanisms behind differences in *k*_600_ for CO_2_ and CH_4_ could have more effect at intermediate wind and intermediate *k*, while at higher wind speeds other factors become more important for gas flux and reduce the *k*_600_ differences. It is worth noting that other processes and factors other than wind influence *k*(10,11) and that the relationships shown in (Figure 2) should not be considered generally valid.

### 4.2 Possible Explanations for Differences in *k*_600_ among Gases

**Figure 3:**
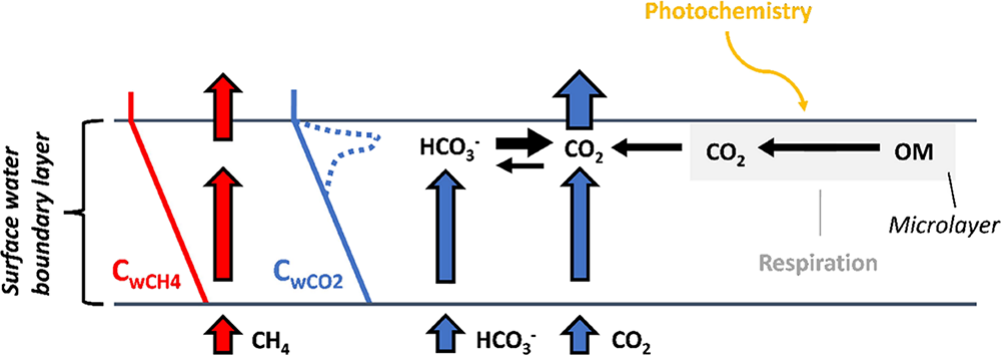
Conceptual figure for potential effect of chemical reactivity and degradation processes in the surface microlayer on concentration gradients of CO_2_ (blue dashed line) in relation to assumed concentration gradients for CO_2_ or CH_4_ without CO_2_ reactivity (solid lines in blue and red, respectively) in waters supersaturated with both CO_2_ and CH_4_. Please note that our intention with the figure is to present processes that contributes to the increase of apparent *k*_600_ for CO_2_ relative to CH_4_, hence relating to the findings from our empirical measurements. Therefore, we chose not to include processes in the figure that have the opposite effect, i.e., enhancing apparent *k*_600_ for CH_4_, e.g., by the contribution of microbubbles or other processes that may be the result of sampling bias.

The mixed results from previous studies, where some observe higher *k*_600CO2_ and others higher *k*_600CH4_, can be due to mechanisms specific to either CH_4_, CO_2_, or to both gases. To highlight how specific mechanisms may influence *k* for CH_4_ and CO_2_, we discuss these mechanisms separately in the subsections below. Mechanisms related to the sampling methods are discussed in the section “Methodological reasons for gas k_600_ differences”. A summary of the mechanisms discussed can be found in Figure 3 and Table 3.

**Table 3:** Overview of Potential Processes for Different Apparent *k*_600_ (Referred to as app. *k*_600_ in Table) Values for CH_4_ and CO_2_^a^

no.	process	effect	explanation	study
1	microbubble flux	app. *k*_600CH4_ > app. *k*_600CO2_	based on hypothesis that microbubbles form that move faster than dissolved gases across the water surface boundary layer and that CH_4_ enters these bubbles to a greater extent than CO_2_. The apparent difference in *k* would in this case result because of a combination of different flux processes combined.	(5,13,18,20)
2	oxic surface water CH_4_ production	app. *k*_600CH4_ > app. *k*_600CO2_	if there is oxic surface CH_4_ production above the depth where surface water CH_4_ and CO_2_ concentrations are measured, the true concentration gradient of CH_4_ is underestimated and apparent *k*_CH4_ will be overestimated.	
3	high surface primary production	app. *k*_600CH4_ > app. *k*_600CO2_	if there is high primary productivity above the depth where surface water CH_4_ and CO_2_ concentrations are measured, the true concentration gradient of CO_2_ is overestimated and apparent *k*_CO2_ will be underestimated.	
4	chemical reactivity	app. *k*_600CH4_ < app. *k*_600CO2_	chemical enhancement of CO_2_ due to equilibration reactions between CO_2_ and bicarbonate can alter the near-surface CO_2_ gradient. In cases for which this process is important, the *k*_600CO2_ should truly exceed *k*_600CH4_.	(7)
5	surface microfilm respiration processes	app. *k*_600CH4_ < app. *k*_600CO2_	for cases with surface films enriched with organic matter where microbial or photochemical processes generate greater respiration and greater CO_2_ concentrations above the depth where surface water CH_4_ and CO_2_ concentrations are measured, the true concentration gradient of CO_2_ is underestimated and apparent *k*_CO2_ will be overestimated.	
6	possible biased gas concentration measurements using equilibrators	app. *k*_600CH4_ > app. *k*_600CO2_	if gas concentration measurements do not fully account for the slower equilibration times for CH_4_, relative to CO_2_, between water and a gas headspace, the CH_4_ concentration will be underestimated and *k*_600CH4_ will be overestimated.	(6,18,20)
7	headspace extraction in waters where *C*_wCO2_ is undersaturated relative to CO_2_ concentrations in the atmosphere	app. *k*_600CH4_ < app. *k*_600CO2_	if not accounting for the chemical equilibration of the carbonate system inside the water sample when calculating *C*_wCO2_ in undersaturated waters, *C*_wCO2_ will be underestimated relative to *C*_wCH4_. This in turn will overestimate apparent *k*_600CO2_.	
8	headspace extraction in waters where *C*_wCO2_ is supersaturated relative to CO_2_ concentrations in the atmosphere	app. *k*_600CH4_ > app. *k*_600CO2_	if not accounting for the chemical equilibration of the carbonate system inside the water sample when calculating *C*_wCO2_ in supersaturated waters, *C*_wCO2_ will be overestimated relative to *C*_wCH4_. This in turn will underestimate apparent *k*_600CO2_.	

a Here *k*_600_ represents the gas transfer velocity normalized to the Schmidt number 600. The explanations are considering processes when *k*_600_ is estimated from combined flux and surface water concentration measurements. Each process in the table is discussed further in the separate paragraphs in Section 4.2. The last column, study, is an attempt trying to link possible effects on apparent *k*_600_ from each specific mechanism presented in Section 4.2 to the studies that are shown in Table 2.

#### 4.2.1 Potential Mechanisms Leading to Higher Estimated *k*_600_ for CH_4_ than for CO_2_

##### 4.2.1.1 Microbubble Flux

Several studies reporting higher *k*_600CH4_ than *k*_600CO2_ considered the possibility of microbubble flux.^5,13,18^ Due to the buoyancy of bubbles, allowing faster transport of dissolved gases, microbubbles could favor the transport of gases with low water solubility, resulting in higher apparent *k* for low-compared to high-soluble gases.

Microbubble flux, enhancing *k* for CH_4_, has also been suggested to be positively linked to the level of CH_4_ supersaturation.^13^ Although dissolved CH_4_ concentrations in freshwater systems are usually supersaturated relative to atmospheric partial pressures (in the order of 2 μatm), the surface water concentrations are far from supersaturated relative to pure CH_4_ (1 atm). Therefore, it is not realistic that the CH_4_ itself should form microbubbles in surface water. However, microbubbles based on other gases can form due to entrainment of air in breaking waves in turbulent aquatic environments^32^ and can remain entrained for several days.^33^ Breaking waves and whitecap formation at wind speeds of 2–3 m s^–1^ has been suggested even at large-fetch systems.^34^ The studies conducted by McGinnis et al.^18^ and Rosentreter et al.^20^ are, to our knowledge, the only freshwater studies that identified relations between potential freshwater microbubble flux and wind speed or current velocity. In contrast, studies conducted by Rantakari et al.^19^ and Prairie and del Giorgio^13^ that suggest higher *k*_600CH4_ than *k*_600CO2_, did not observe such patterns. Studies by Beaulieu et al.^5^ and Paranaíba et al.,^6^ also suggesting higher *k*_600CH4_ than *k*_600CO2_, did not test for the above relationships. When it comes to our study, we did not experience any conditions with breaking waves or whitecap formation while sampling, thus limiting the potential of microbubble flux contribution.

Microbubbles, regardless of the formation mechanism, could transport all supersaturated gases, i.e., not only CH_4_ but also CO_2_ and other gases. The relative microbubble transport rates could be solubility dependent as suggested (favoring the transport of CH_4_ over CO_2_), but given the higher transfer rates of supersaturated CO_2_ from water into a headspace (Figure S4), significant transport of CO_2_ or other soluble gases via microbubbles cannot be excluded. Moreover, Beaulieu et al.^5^ observed no significant differences between *k* for CH_4_ and nitrous oxide (N_2_O) (t-test, *p* = 0.52), although solubility of N_2_O is similar to CO_2_ and would be expected to have lower *k* than CH_4_ if microbubble flux was occurring and was solubility dependent.

Our results may indicate that *k*_600CH4_ relative to *k*_600CO2_ decrease with CH_4_ supersaturation (Section 3; Figure S2b), and our calculations on the potential microbubble flux (*F*_mb_), according to Prairie and del Giorgio^13^ (Text S2), show negligible effects on the enhancement of *k* for CH_4_. This does not support previous suggestions on microbubble formation and gas transport in boreal lakes^13^ and shows that the relationship between microbubble flux and enhanced *k* for CH_4_ needs further consideration. Based on the above-mentioned discussion, it is clear that the microbubble hypothesis, as an explanation for enhanced *k* for CH_4_ relative to *k* for CO_2_, is not generally applicable and instead context dependent. The extent to which microbubbles influence apparent *k*_600_ for different gases is therefore still an open question. Some of the alternative explanations outlined below may in many situations be more likely when explaining differences in *k*_600_ among gases.

##### 4.2.1.2 Oxic Surface Water CH_4_ Production

CH_4_ can be produced in waters under oxic conditions,^35,36^ and there is support for such production associated with photosynthesis by cyanobacteria and other mechanisms. The extent of this surface water CH_4_ production is debated, but if it occurs close to the water surface, it could contribute to CH_4_ being formed in the surface boundary layer, making the real concentration gradient steeper than measured from deeper sampling, and leading to overestimating the apparent *k*_600CH4_ when estimating *k* from concentration measurements below the boundary layer.

##### 4.2.1.3 High Surface Primary Production

Under some conditions, e.g., severe light limitation caused by algal blooms, it is possible (albeit unlikely—see “Surface microfilm processes” below) that the majority of the primary production may be restricted to the uppermost (few centimeters) of the water, resulting in limited productivity below this layer.^37^ This in turn could lead to elevated CO_2_ consumption near the water surface, and *C*_wCO2_ sampled some cm or deeper below the water surface may not represent the real *C*_wCO2_ driving the CO_2_ gas exchange. In such cases, *C*_wCO2_ could be overestimated yielding underestimation of *k*_600CO2_, resulting in a higher apparent *k*_600CH4_ than *k*_600CO2_. This explanation illustrates a case of method bias if gas concentration measurements do not represent the actual concentration gradient shaping the gas fluxes.

#### 4.2.2 Potential Mechanisms Leading to Higher *k*_600_ for CO_2_ than for CH_4_

##### 4.2.2.1 Chemical Reactivity

In contrast to dissolved CH_4_, CO_2_ has two ways to pass the water-side boundary layer at the water-atmosphere interface. One way, which is shared with other gases, is via molecular diffusion. In addition, CO_2_ can react with the water and form bicarbonate or carbonate ions. Accordingly, a part of the CO_2_ can diffuse through the water boundary layer as bicarbonate (Figure 3). This generates a second way for CO_2_ to cross the water-atmosphere interface that is not shared by dissolved CH_4_. At high pH, which is common in lakes with high primary productivity and low inorganic carbon concentrations, this is well acknowledged as chemical enhancement.^15,21^ However, at low pH, when the carbonic acid equilibrium system favors a dominance of CO_2_ as the net result of equilibrium reactions, there is still a continuous formation of bicarbonate that could potentially contribute to the transport of CO_2_ across the surficial boundary layer. As a part of this mechanism and in the case with CO_2_-supersaturated waters, loss of CO_2_ through emissions to the atmosphere will shift the inorganic carbon equilibrium balance in the surface water boundary layer to convert more bicarbonate to CO_2_, which leads to a higher resupply of CO_2_ in the layer where the actual loss to the atmosphere occurs. This dual mechanism for passage across the diffusive boundary layer differs from gases not reacting with the water (such as CH_4_). Thereby, water samples taken below the boundary layer may underestimate the concentration gradient of CO_2_ and in turn overestimate *k*_600_ for CO_2_ in relation to gases such as CH_4_ (Figure 3). A higher reactivity for CO_2_ compared to CH_4_ in the surficial boundary layer, that is not limited to conditions of high pH, might explain the higher apparent *k*-values of CO_2_ that we observed during conditions of CO_2_ supersaturation, and similar observations being consistent with this explanation have also been obtained in CO_2_-supersaturated parts of estuaries.

##### 4.2.2.2 Surface Microfilm Processes Stimulating CO_2_ Production

The surface microfilm at the air–water interface of water bodies is often enriched in nutrients, particulate and dissolved carbon, phytoplankton, and microbes.^38−40^ This is a zone where photochemical processes can degrade dissolved organic matter to CO_2_ and labile compounds, providing additional substrate for microbes^41−44^ (Figure 3). This is also a zone where photoinhibition may reduce primary production^37,45,46^ and where buoyant particles aggregate and are respired, which enhances net production of CO_2_ (Figure 3). Hence, *C*_wCO2_ measurements a few centimeters into the water may underestimate the *C*_wCO2_ and in such cases *k*_600CO2_ will be overestimated and the apparent result would be that *k*_600CO2_ is greater than *k*_600CH4_.

Biological and chemical processes in ocean surface microlayers have been suggested to influence *k*_600_,^14,47−49^ but to our knowledge their effect on *k*_600_ in inland waters is largely unknown. A study using FCs in the tropical Atlantic Ocean found mismatches in *k*_600_ between CH_4_, carbon monoxide (CO), N_2_O, and hydrogen (H_2_) and concluded that microbial gas consumption within the surface layer film was the only plausible explanation for such differences in *k*_600_.^14^ Frost^47^ came to the same conclusion when he identified a mismatch with up to 8% higher *k*_600_ for CH_4_ compared to sulfur hexafluoride (SF_6_) in the North Sea, and results were corroborated by Upstill-Goddard et al.,^50^ who replicated similar patterns in a controlled experiment by adding methanotrophs to the surface microlayer film. In the subtropical Atlantic, Calleja et al.^51^ found that *k*_600_ for CO_2_ was controlled by microbial metabolism in the uppermost (<2 cm) water layer, with sevenfold and tenfold differences in respiration and gross primary production, respectively, compared to the mixed photic layer below. If the above findings in ocean systems are valid for lakes, microbial communities within the uppermost water layer could potentially alter the concentration of CH_4_ or CO_2_ in lakes and bias our *k* determination method. It might explain the contrasting patterns found in different systems, where some studies indicate enhanced *k*_600CH4_ and some show enhancement of *k*_600CO2_.

#### 4.2.3 Methodological Reasons for Gas *k*_600_ Differences

Discrete sample headspace analysis is associated with a risk of introducing systematic error into calculations if chemical equilibration of the carbonate system inside the sample vial is not accounted for. Koschorreck et al.^17^ found that such bias could lead to error, underestimating *C*_wCO2_ by a factor of 3 in highly undersaturated waters when using atmospheric air for the equilibration, but overestimating *C*_wCO2_ by less than 5% in supersaturated (>1000 μatm) waters. Bias is reduced if a high water to air volume ratio is used in the sampling syringe. We accounted for this error when calculating *C*_wCO2_, but bias would otherwise only be ∼2% due to high supersaturation in our lakes and a water to air volume ratio of ∼19 when sampling. Nevertheless, it is possible that the findings from Koschorreck et al.^17^ could influence the *k*_600_-ratios (CO_2_/CH_4_) presented in Table 2 if not considered in all studies.

When using flow-through equilibrators, a mismatch between *k* for CO_2_ and CH_4_ could increase if differences in gas equilibration times are not accounted for.^16^ The equilibration time for CH_4_ is considerably longer than for CO_2_ due to the greater supersaturation of CH_4_ in water and its lower solubility, and thereby the greater proportional mass transport of CH_4_ is needed from the water to the gas phase to reach equilibrium (Figure S4). Accordingly, if measurements of *C*_wCH4_ are not fully equilibrated, this will underestimate *C*_wCH4_ and consequently result in overestimation of *k*_600_ for CH_4_.

Regardless of choosing equilibrator or headspace extraction approaches, the depth of the water sampling is critical, as highlighted above. Potential explanations for the differences between apparent *k*_600_ for CO_2_ and CH_4_ could be attributed to methodological inconsistency regarding the depth where water samples are taken to measure the gas concentration due to gradients in the very near-surface water. From this perspective, it could be argued that a more accurate way to estimate the surface water gas concentrations driving the flux may be to use long-term FC equilibrations, designed to make in situ chamber headspace gas concentrations reflect the gas concentrations in the uppermost water layer. Further exploration of such chambers designed for quickest possible equilibration rates (e.g., large surface area to volume ratio and constant water renewal under the chamber^28^) and evaluating their pros and cons would be of interest to improve future *k* measurements.

Another methodological aspect regards the common temporal mismatch between flux measurements (integrating gas transport over 10–30 min) and instantaneous snap-shot concentration measurements or from the delay in the equilibration of chambers. This contributes uncertainty to *k*-estimates as the flux and concentration data used to calculate *k* represents different time periods. The direction of this bias depends on how gas concentrations in the water change during the flux measurement. This time-mismatch may therefore not cause a systematic bias on *k*_600_ differences among gases if considering many measurement periods and systems. However, reducing the related *k* uncertainty needs further consideration by, e.g., combining flux measurements with repeated or continuous concentration measurements during the whole flux measurement period or optimized FCs for rapid equilibration.

### 4.3 Future Considerations and Implications

It is imperative to understand controls on *k*_600_ to accurately estimate gas fluxes from surface waters. Our results from four boreal lakes add to the growing literature which indicates that current assumptions made when calculating *k*_600_ from measurements designed for other gases, do not hold when tested in situ. Additional mechanisms than those outlined here may be possible. For example, variability in *k* values for CO_2_ and CH_4_ may also depend on conditions within the water column, and exploring potential patterns in *k*_600CH4_ to *k*_600CO2_ versus, e.g., local hydrodynamics influenced by heating, cooling, and wind speed and direction would be of interest. Clearly, several explanations to observed differences in *k*_600_ among gases exist that can interact and differ in relative importance among studies. This is a matter of concern as *k*-dependent gas exchange models are widely used in regional studies due to the perceived simplicity of collecting water samples to estimate gas concentration and modeling *k* to derive fluxes in comparison with making actual flux measurements. Many such studies, incorporating *k*-dependent gas exchange models, are used in global upscaling of fluxes, and the discrepancy-ranges observed in *k*_600_ seem large enough to substantially affect aquatic greenhouse gas emissions estimates. Future studies should be designed to address potential biases in gas concentration measurements and account for the possible mechanisms that may affect *k* differently for CO_2_ and CH_4_. Before these challenges are addressed, attempts to convert *k* from one gas to another based solely on physical properties may not be reliable beyond controlled laboratory conditions, and in situ empirical assessments of *k* for each gas of interest remain important for accurate flux assessments.

## Supporting Information Available

Gas calculations for CO_2_ and CH_4_ in lake systems SOR, BOL, and SOD (Text S1) and supplemental method description of microbubble flux calculations (Text S2), with figures showing the in situ sampling setup using UGGA and flux chamber for measuring fluxes of CH_4_ and CO_2_ (Figure S1), the relationship between measured, lake dissolved CO_2_ and CH_4_ concentrations, and lake CO_2_ and CH_4_ fluxes (Figure S2), the measured *k*_600_ ratio (CO_2_/CH_4_) and CO_2_ saturation, and CH_4_ saturation (Figure S3), and gas specific equilibration time measurements for CO_2_ and CH_4_ in an aquatic environment (Figure S4) (PDF)
Source data contain data needed to reproduce results in the manuscript and include in situ measurements of the apparent *k*_600_ for CO_2_ and CH_4_, wind speeds approximated at 10 m height (U_10_), apparent *k*_600_ ratio (CO_2_/CH_4_), dissolved CO_2_ and CH_4_, gas fluxes of CO_2_ and CH_4_, and apparent ratio of dissolved gases in the surface waters versus the above lying atmosphere (*C*_w_/*C*_air_) for CO_2_ and CH_4_, respectively (XLSX)
