# Supplementary material for: Higher Apparent Gas Transfer Velocities for CO2 Compared to CH4 in Small Lakes
Source: Environ Sci Technol. 2023 May 30;57(23):8578–87. doi: 10.1021/acs.est.2c09230 (PMC10269337; doi:10.1021/acs.est.2c09230)
Supplement: Supplementary file 1 — es2c09230_si_001.pdf [file es2c09230_si_001.pdf]

Supporting information for

Higher apparent gas transfer velocities for CO_2 compared to CH_4 in small lakes

Gustav Pajala[a*], David Rudberg[a], Magnus Gålfalk[a], John Michael Melack[b,c], Sally Macintyre[b,c,d], Jan Karlsson[e], Henrique Oliveira Sawakuchi[a], Jonathan Schenk[a], Anna Sieczko[a], Ingrid Sundgren[a], Nguyen Thanh Duc[a], David Bastviken[a*]

[a]Department of Thematic Studies – Environmental Change, Linköping University, 58183 Linköping, Sweden.

[b]Department of Ecology, Evolution and Marine Biology, University of California, Santa Barbara, California, USA

[c]Earth Research Institute, University of California, Santa Barbara, California, USA

[d]Marine Science Institute, University of California, Santa Barbara, California, USA

[e]Climate Impacts Research Centre, Department of Ecology and Environmental Sciences - Umeå University, Umeå, Sweden

*Corresponding authors at: Department of Thematic Studies – Environmental Change, Linköping University, 58183 Linköping, Sweden.

*E-mail address: Gustav.pajala@liu.se and david.bastviken@liu.se

Contents

Text S1. Calculation of gas fluxes in SOR, BOL, and SOD.

Text S2. Calculation of microbubble flux contribution.

Figure S1. Relationship between CO_2 and CH_4 water concentrations (panel a) and gas fluxes (panel b).

Figure S2. Relationship between the k_{600} ratio for CO_2 and CH_4 versus CO_2 saturation (panel a) and CH_4 saturation (panel b).

Figure S3. CO_2 and CH_4 gas measurement over time using the Ultra-Portable Greenhouse-gas analyzer (UGGA).

Figure S4. Fieldwork setup using a flux chamber (FC) connected to the UGGA for gas measurements.

Number of figures: 4

Number of equations: 2

Number of pages: 9

Text S1 (calculation of gas fluxes in SOR, BOL, and SOD)

Linear regressions for calculating the gas accumulation rates for CO_2 and CH_4, representing the increase in mole fraction per time unit (ppm d^{-1}), were made in Excel using the function SLOPE for time periods of 40 to 100 seconds. R^2 values for the linear regressions were determined using the function RSQ where linear regressions having and $R^2 > 0.7$ were used as final gas accumulation rates. To convert gas accumulation rates to gas fluxes F_{gas} (mmol m^{-2} d^{-1}), we applied the ideal gas law and divided with the chamber area, see Equation (S1),

$$F_{\mathrm{gas}} = \frac{\mathrm{ppm}}{t} * \frac{(PV*0.0001)}{RTA} \qquad \text{(S1)}$$

where the barometric pressure P (hPa) was obtained from the Swedish Meteorological and Hydrological Institute (SMHI) MESAN model, V represents the chamber headspace volume (m^3), 0.0001 was as used as a conversion factor to convert the gas accumulation rates (ppm d^{-1}) and the barometric pressure (hPa) to (pa d^{-1}), R is the ideal gas constant, 8.314 (m^3 Pa K^{-1} mol^{-1}), T represents the water temperature (K), and A represents the chamber area (m^2).

Text S2 (calculation of microbubble flux)

Microbubble mediated flux F_{mb} (mol m^{-2} d^{-1}) was calculated based on the equation from Prairie & Del Giorgio[1], using the apparent k_{600} calculated for CO_2 corrected to CH_4, according to equation (S2) below:

$$F_{mb} = F_{CH4} - k_{600CO2} * kH[p_{CH4water} - p_{CH4air}] \quad \text{(S2)}$$

where F_{CH4} is the observed CH_4 flux (mol m^{-2} d^{-1}), k_{600CO2} is the apparent k_{600} for CO_2 normalized to a Schmidt number of 600 (m d^{-1}), kH is the temperature corrected Henry's law constant (M atm^{-1}), $p_{CH4water}$ is the partial pressure of CH_4 in equilibrium with the surface water concentration (atm) and p_{CH4air} is the partial pressure in the flux chamber at sampling (atm).

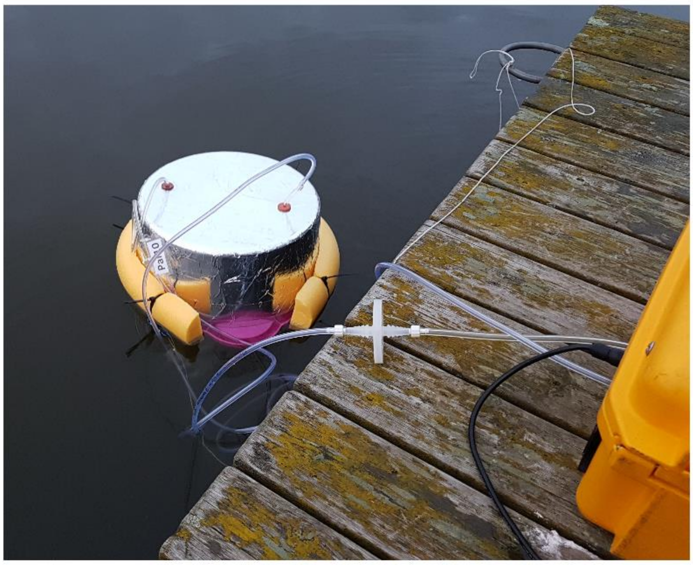

Figure S1. Gas sampling setup using the UGGA in SOR, BOL, and SOD (photo by G. Pajala).

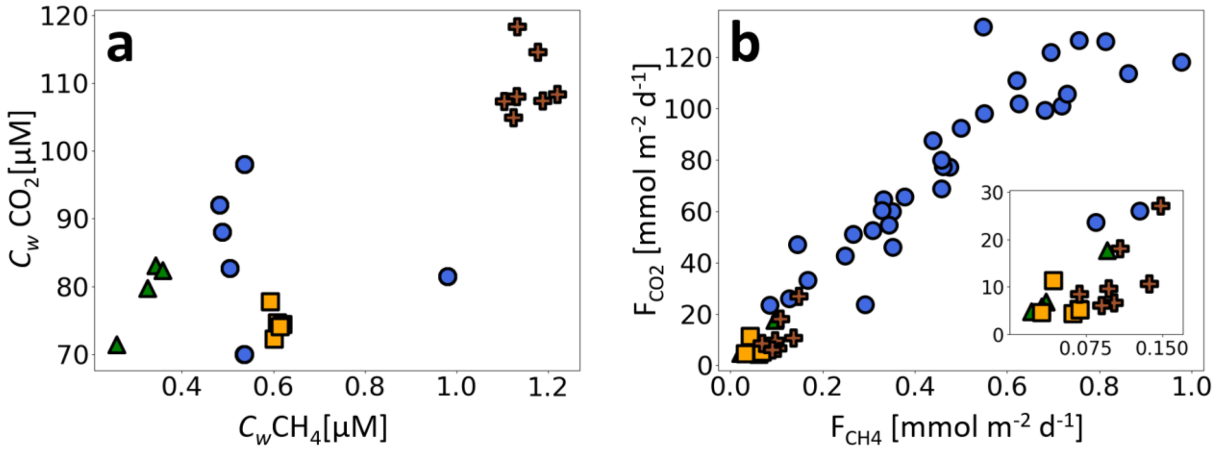

Figure S2. Relations between **(a)** surface water concentrations of CH_4 and CO_2 (C_{wCH4} and C_{wCO2}) and **(b)** diffusive fluxes of CH_4 and CO_2. Lake observations are represented by blue circles (OBJ), green triangles (BOL), orange squares (SOD) and brown crosses (SOR). The inset graph in panel B shows a zoom of the low flux observations.

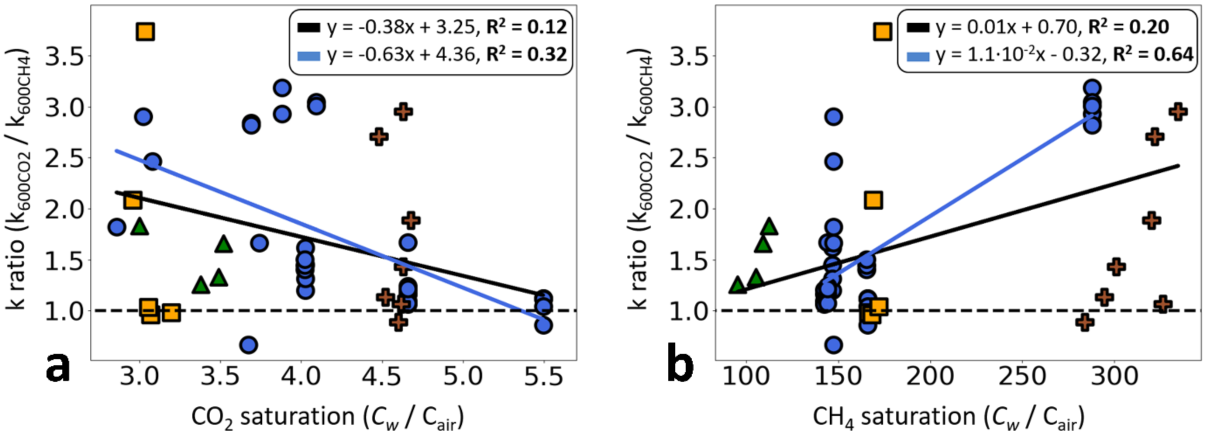

Figure S3. Relation between k_{600CO2}/k_{600CH4} ratio and **(a)** CO_2 saturation and **(b)** CH_4 saturation. Lake observations are represented by blue circles (OBJ), green triangles (BOL), orange squares (SOD) and brown crosses (SOR). Regressions are calculated for all lakes combined (black line) and for OBJ (blue line). The dashed line denotes the separation between higher (above) and lower (below) k_{600CO2} than k_{600CH4}.

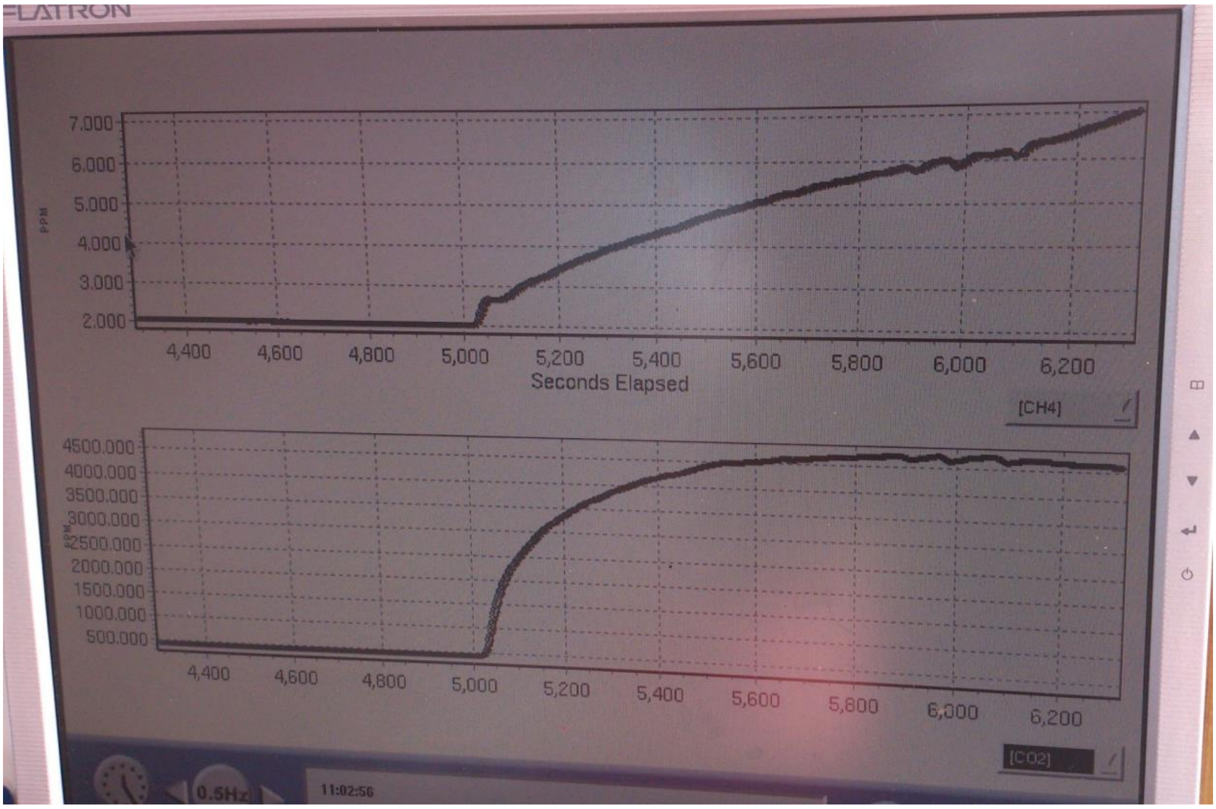

Figure S4. Screenshot from continuous CH_4 (top) and CO_2 (bottom) measurement in a gas equilibrator used in aquatic environments, showing differences in equilibration time among gases. In this equilibrator, CO_2 equilibrium was reached after approximately 8 minutes, whereas CH_4 still changed linearly after 20 minutes without any sign of approaching equilibrium (photo by D. Bastviken).

References

1. Prairie, Y.; del Giorgio, P., A new pathway of freshwater methane emissions and the putative importance of microbubbles. *Inland Waters* **2013,** *3* (3), 311-320.
